# The Potential Applications of Real-Time Monitoring of Water Quality in a Large Shallow Lake (Lake Taihu, China) Using a Chromophoric Dissolved Organic Matter Fluorescence Sensor

**DOI:** 10.3390/s140711580

**Published:** 2014-06-30

**Authors:** Cheng Niu, Yunlin Zhang, Yongqiang Zhou, Kun Shi, Xiaohan Liu, Boqiang Qin

**Affiliations:** 1 Taihu Lake Laboratory Ecosystem Research Station, State Key Laboratory of Lake Science and Environment, Nanjing Institute of Geography and Limnology, Chinese Academy of Sciences, Nanjing 210008, China; E-Mails: hhuniucheng@163.com (C.N.); yohnchou917251@126.com (Y.Z.); kshi@niglas.ac.cn (K.S.); liuxiaohan7763@163.com (X.L.); qinbq@niglas.ac.cn (B.Q.); 2 University of Chinese Academy of Sciences, Beijing 100049, China

**Keywords:** chromophoric dissolved organic matter, dissolved organic carbon, fluorescence, real-time monitoring, water quality parameters

## Abstract

This study presents results from field surveys performed over various seasons in a large, eutrophic, shallow lake (Lake Taihu, China) using an *in situ* chromophoric dissolved organic matter (CDOM) fluorescence sensor as a surrogate for other water quality parameters. These measurements identified highly significant empirical relationships between CDOM concentration measured using the *in situ* fluorescence sensor and CDOM absorption, fluorescence, dissolved organic carbon (DOC), chemical oxygen demand (COD) and total phosphorus (TP) concentrations. CDOM concentration expressed in quinine sulfate equivalent units, was highly correlated with the CDOM absorption coefficient (*r*^2^ = 0.80, *p* < 0.001), fluorescence intensities (Ex./Em. 370/460 nm) (*r*^2^ = 0.91, *p* < 0.001), the fluorescence index (*r*^2^ = 0.88, *p* < 0.001) and the humification index (*r*^2^ = 0.78, *p* < 0.001), suggesting that CDOM concentration measured using the *in situ* fluorescence sensor could act as a substitute for the CDOM absorption coefficient and fluorescence measured in the laboratory. Similarly, CDOM concentration was highly correlated with DOC concentration (*r*^2^ = 0.68, *p* < 0.001), indicating that *in situ* CDOM fluorescence sensor measurements could be a proxy for DOC concentration. In addition, significant positive correlations were found between laboratory CDOM absorption coefficients and COD (*r*^2^ = 0.83, *p* < 0.001), TP (*r*^2^ = 0.82, *p* < 0.001) concentrations, suggesting a potential further application for the real-time monitoring of water quality using an *in situ* CDOM fluorescence sensor.

## 1. Introduction

Lakes and reservoirs are abundant natural systems that provide a wide range of essential ecosystem services, ranging from drinking water and food to transportation and recreation [1]. In addition, lakes and reservoirs are important sentinels, integrators, and regulators of climate change [2]. However, in the past few decades, many lakes and reservoirs have undergone increasing eutrophication, water quantity decrease and water quality deterioration due to the dual pressures of anthropogenic activities and climate change [3–5]. As a result, some lakes and reservoirs have experienced phytoplankton blooms and drinking water crises. For example, in late May 2007, a drinking water crisis occurred in Wuxi, Jiangsu Province, China, following a massive bloom of the toxin-producing cyanobacteria *Microcystis spp.* in Lake Taihu, causing approximately two million people to be without drinking water for at least a week [3].

Therefore, high-frequency accurate and precise measurements of lake water quality are vital to investigate the causes of water quality variability on hourly to interannual timescales and to understand the mechanisms through which water quality affects lake ecosystem structure and function. In addition, effective and fast water quality monitoring will improve water quality and water resource management for management administrations. However, in the past decades, field measurements for water quality evaluation have typically depended upon costly time- and labor-intensive on-site sampling and data collection and transport to land-based or shipboard laboratories for evaluation. While these research and monitoring efforts are episodically intensive, they generally have been too limited, both temporally and spatially, to adequately address factors affecting the development of events such as harmful algal blooms, oxygen depletion, and contamination from chemical plants. Satellite-based remote sensing using the Sea-viewing Wide Field-of-view Sensor (SeaWiFS), Moderate Resolution Imaging Spectroradiometer (MODIS), Medium Resolution Imaging Spectrometer (MERIS), and Landsat ETM can offer high spatial resolution, from several meters to kilometers, for partial water quality parameters. However, remote sensing is typically discontinuous (*i.e*., the revisit period is between several hours to more than ten days), which is not compatible with the intrinsic short-term, rapid variability of water quality parameters.

For the reasons mentioned above, increasing attention has been paid to methods that are able to sensitively and continuously detect and quantify lake water quality parameters. Recent advances in communication and sensor technology have catalyzed progress in on-line real-time monitoring capabilities for water quality. However, few sensors, such as dissolved oxygen, chlorophyll *a* and chromophoric dissolved organic matter (CDOM) sensors, can be developed for sub-surface *in situ* measurements on floats, robots, or gliders [6–8]. Therefore, various authors have published data to calibrate and validate empirical predictive models that predict other water quality parameters using *in situ* sensor data [9–11].

CDOM is an important optically active substance in aquatic ecosystems and affects UV light penetration, nutrient cycling, ecosystem productivity, heavy metal transport, contaminant availability and drinking water quality [12]. In addition, the fluorescent fraction of CDOM, referred to as CDOM fluorescence or FDOM, has widely been used to trace CDOM amounts, sources, composition, and as a surrogate for dissolved organic carbon (DOC), biological oxygen demand (BOD) and chemical oxygen demand (COD) concentrations [11–13]. Several companies manufacture *in situ* CDOM fluorometers using excitation and emission at 370 and 460 nm, respectively (e.g., Wetlabs/CDOM WETStar; Turner/Cyclops 7; Trios/micro Flu CDOM; and SeaPoint/SUVF) [8,10,14,15], for a range of applications in freshwater and coastal systems.

At present, *in situ* CDOM sensors have been used in many different environments (e.g., wetlands, forested watersheds, agricultural watersheds and tidal marshes) to provide a relatively inexpensive, high-resolution proxy for DOC concentration and, in some cases, other related biogeochemical variables such as methyl mercury (MeHg) concentration [9,10,16]. However, until now, the potential application of a CDOM fluorescence real-time monitoring sensor for estimating other water quality parameters has not been fully explored.

Lake Taihu, the third largest freshwater lake in China, lies in the east of the country at 30°55′40″–31°32′58″N and 119°52′32″–120°36′10″E. The total area of the lake is 2427.8 km^2^, and the water surface area is 2338.1 km^2^; the mean water level is 3.0 m above sea level [17]. As a typical large eutrophic lake, complex river organic pollution inputs and frequent algal blooms have caused a high CDOM concentration and even areas of black water, which are characterized as hypoxic, malodorous and harmful areas that threaten drinking supplies and ecosystem security [3,18,19]. Therefore, it is urgent to continuously monitor CDOM via real-time high-frequency sensors. In light of the success of previous studies, this study investigated the utility and feasibility of using a fluorescence sensor to monitor CDOM and organic matter concentrations within a large eutrophic shallow lake. Subsequently, in this study, the focus was on developing predictive relationships for lake water quality parameters such as DOC, COD, total nitrogen (TN) and total phosphorus (TP) concentrations based on measurements from a real-time *in situ* CDOM sensor.

Therefore, the objectives of our study were to: (1) determine whether correlations exist between CDOM concentration measured using *in situ* fluorescence sensor and laboratory CDOM absorption coefficients and fluorescence; (2) determine whether CDOM concentration could be correlated with DOC concentration; and (3) expand the potential application of an *in situ* fluorescence sensor to estimate COD and TP concentrations based on the empirical correlations between CDOM absorption coefficients and COD and TP concentrations.

## 2. Materials and Methods

### 2.1. Study Area and Sampling Sites

**Figure 1.**
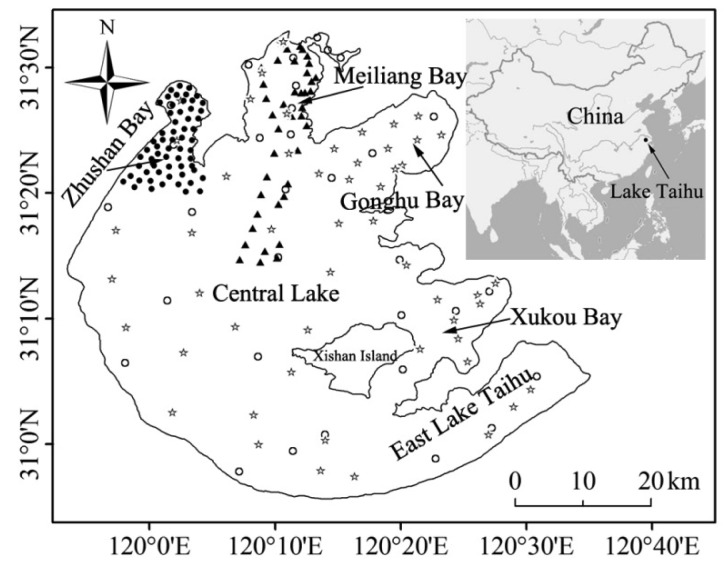
Distribution of sampling sites in Lake Taihu from five cruises. (1) In August 2004, 40 sampling sites were distributed in Meiliang Bay and Lake Center (▲); (2) In February and May 2010, 32 sampling sites (○) were scattered throughout the lake; (3) In August 2013, 53 sampling sites (☆) were scattered throughout the lake; (4) In April 2014, 61 sampling sites were distributed in Zhushan Bay (●).

The dataset in this study, used to investigate *in situ* CDOM sensor utility and feasibility and develop predictive relationships, was composed of 218 water samples collected from five cruises. The sampling sites (Figure 1) were distributed around the lake in August, 2013 (☆, 53 sites) and in Zhushan Bay in April, 2014 (●, 61 sites) during two cruises. The collected data included *in situ* fluorescence sensor measurements and water samples collections to measure CDOM absorption, fluorescence and DOC concentration. The sampling sites were distributed in Meiliang Bay, lake center in August, 2004 (▲, 40 sites) and around the lake in February and May, 2010 (○, 32 × 2 = 64 sites); these cruises only collected water samples to measure CDOM absorption and COD, TN and TP concentrations. The dataset composed of 114 water samples from 2013 and 2014 was used to investigate the utility and feasibility of a CDOM sensor to monitor CDOM absorption, fluorescence and DOC concentration. The dataset composed of 104 water samples from 2004 and 2010 was used to develop predictive relationships between COD, TN and TP concentrations and CDOM absorption coefficient to expand the potential application of a CDOM sensor.

### 2.2. Water Samples Collection and In Situ CDOM Measurement

Water sample collection and field monitoring were conducted at a depth of 1.0 m. Surface water samples were collected in 2 L acid-washed bottles and held on ice while in the field. CDOM concentration was measured using an *in situ* CDOM fluorescence sensor (TRIOS GmbH, Rastede, Germany) at the depth of 1.0 m with the excitation and emission wavelengths at 370 and 460 nm (e.g., 20 nm of full width at half maximum), respectively. The measuring range of *in situ* CDOM fluorescence sensor was 0–200 μg/L with a sensitivity of 0.2 μg/L. The maximal sampling depth was 500 m and the operating temperature was between −10 °C and +50 °C. The fluorometer was monitored with a computer via the power supply, and the telemetry monitoring unit was checked via a digital connection. The fluorescence was measured for 5 min with a sampling interval of 5 s at every site. The average value of all recordings in 5 min was considered the CDOM concentration of this site. The fluorescence sensor was not equipped with a filtering device to remove the effect of particles. CDOM concentration measured by the fluorescence sensor was calibrated and normalized via quinine sulfate units (μg/L).

### 2.3. UV-Vis Absorption Measurements

Absorption spectra were obtained between 200 and 800 nm at 1 nm intervals using a UV–Vis spectrophotometer (UV–2550PC, Shimadzu-China, Suzhou, China) with matching 5.0 cm quartz cells. The slit width was set 1 nm, and the wavelength scan rate was 210 nm/min. Milli–Q water was used in the reference cell. Absorbance measurements at each wavelength were baseline corrected by subtracting the absorbance at 700 nm. Absorption coefficients were calculated by multiplying the corrected absorbance by 2.303/*r*, where *r* is the cuvette path length in m [20]. Considering that the photometric accuracy of the Shimadzu UV–2550PC spectrophotometer was 0.002 and the cuvette path length was 0.05 m, the precision of the CDOM absorption coefficient was 0.092 m^−1^. In this study, we express the abundance of CDOM using the absorption coefficient (m^−1^) at a wavelength of 370 nm, which was widely used in other similar studies [8,10,21] because the *in situ* CDOM sensor was developed with excitation and emission at 370 and 460 nm; however, the absorption coefficient at 254 nm or 350 nm is widely used in DOM and CDOM studies [11,20,22].

### 2.4. Excitation Emission Matrix Spectra Fluorescence Measurement

The excitation emission matrix spectra (EEMs) fluorescence of CDOM were measured in cells with a 1.0 cm path length using a Hitachi (Tokyo, Japan) F-7000 fluorescence spectrometer with a 700-voltage xenon lamp at room temperature (20 ± 2 °C). The scanning ranges were 200–450 nm for excitation and 250–600 nm for emission. Readings were collected in ratio mode (S/R) at 5 nm intervals for excitation wavelength and 1 nm intervals for emission wavelength using a scanning speed of 2400 nm/min. The bandpass widths were 5 nm for both excitation and emission.

Water Raman scatter peaks were eliminated by subtracting a Milli-Q water blank of the EEMs from the sample EEMs. The spectra were corrected for instrument response according to the procedure recommended in the Hitachi Instruction Manual, and the measured CDOM absorbance corrected to a 1.0 cm path length was used to correct the measured EEMs to eliminate the inner-filter effect [23,24]. In brief, the EEMs were corrected for absorbance by multiplication of each value in the EEMs with a correction factor, based on the premise that the mean path length of the absorption of the excitation and emission light was 1/2 of the cuvette length [25].

Daily variations in fluorescence intensity were calibrated and normalized in quinine sulfate units (QSU), where 1 QSU was the maximum fluorescence intensity of 0.01 mg/L of quinine (qs) in 1 N H_2_SO_4_ at the excitation wavelength (Ex; nm)/emission wavelength (Em; nm) = 350/450 [26]. Rayleigh scatter effects were removed from the dataset by adding zero to the EEMs in the two triangle regions (Em ≤ Ex + 20 nm and ≥ 2Ex − 10 nm) [27].

### 2.5. Calculation of Fluorescence Indices

Several indices have been widely used to define and classify the CDOM characteristics, composition and sources. In this study, we introduced the fluorescence index and the humification index (HIX: *FI*_254_) to determine whether CDOM concentration measured using *in situ* fluorescence sensor could replace these indices to characterize CDOM. To distinguish the sources of isolated aquatic fulvic acids, a fluorescence index (*FI*_370_) was presented based on the ratio of the fluorescence intensity at an emission wavelength of 450 nm to that at 500 nm, both excited at 370 nm by McKnight *et al.* [25]. Cory and McKnight modified this metric to be the ratio of the fluorescence intensity at an emission wavelength of 470 nm to that at 520 nm [28]; their metric was used in this study. *FI*_254_ is defined as the ratio between the integrated fluorescence intensity between 435 and 480 nm to that between 300 and 345 nm, both excited at 254 nm [29]. In the present study, we used the excitation wavelength of 255 nm with the same emission wavelength ranges because EEMs were measured for excitation wavelength every 5 nm.

### 2.6. Water Quality Parameter Measurement

Water quality parameters including DOC, COD, TN, and TP in the water column were determined according to the procedures for “Standard Methods for the Examination of Water and Wastewater” [30]. Specifically, DOC was filtered through a pre-combusted 0.7 μm GF/F filter (Whatman, city, state abbrev if US, country) and then measured by a total organic carbon analyzer (TOC-L CPH, Kyoto, Shimadzu) using high-temperature catalytic oxidation (HTCO). COD concentration was measured by titration with acidic potassium permanganate. TP concentration was determined photometrically following the molybdenum blue method in unfiltered lake water after digestion with alkaline potassium persulphate (K_2_S_2_O_8_ + NaOH). TN concentration was analyzed after digestion at a wavelength of 210 nm.

### 2.7. Statistical Analysis

Regression and correlation analyses were performed using SPSS 17.0 software (SPSS Inc., Chicago, CA, USA). The correlations were evaluated using *p*-values and were considered significant when *p* < 0.05. Contour maps showing the spatial distributions of CDOM concentration measured using *in situ* fluorescence sensor were generated in ArcMap 10.0 based on the interpolation method of kriging.

## 3. Results and Discussion

### 3.1. Correlation between CDOM concentration and Absorption, Fluorescence

**Figure 2.**
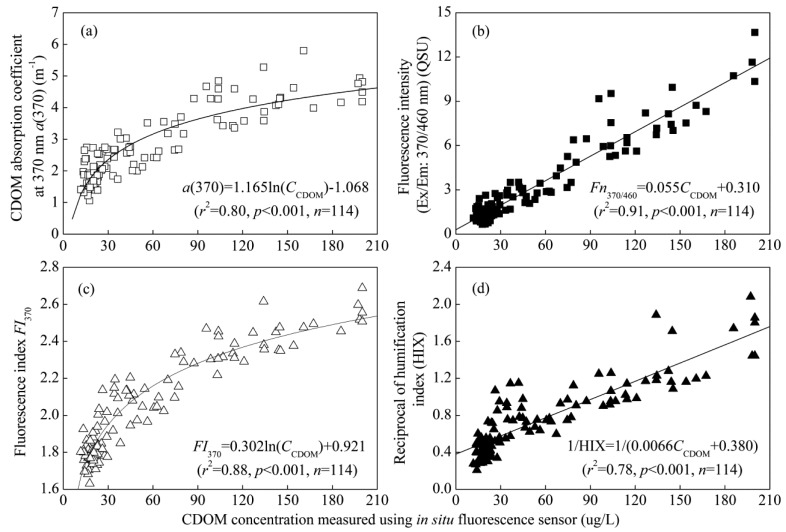
Correlations between CDOM concentration measured using *in situ* fluorescence sensor and absorption coefficient at 370 nm, *a*(370) (**a**), the fluorescence intensity at the excitation/emission wavelengths of 370/460 nm (**b**), the fluorescence index, *FI*_370_ (**c**), the humification index, HIX (**d**) in filtered water in Lake Taihu.

The CDOM absorption coefficient at 370 nm *a*(370) was chosen to describe changes in CDOM quantity. This wavelength is the maximum of the excitation band of the *in situ* CDOM sensor. There was a highly positive correlation between CDOM concentration measured using *in situ* fluorescence sensor and *a*(370) (Figure 2a). Regression analysis between CDOM concentration measured using *in situ* fluorescence sensor and the fluorescence intensities of filtered water was also performed to check the consistency of the fluorescence of the *in situ* sensor and laboratory fluorescence spectrometers. The coefficient of determination of the linear relationship was 0.91, and both slope coefficient and intercept were statistically significant at the confidence level of *p* < 0.001 (Figure 2b) indicating a high degree of consistency. The small intercept (0.310) of the regression equation can be interpreted as the fluorescence signal of CDOM adsorbed on the particles which had not been measured using the *in situ* CDOM sensor. Of all the empirical correlations, the determination coefficient between CDOM concentration and laboratory CDOM fluorescence is the highest because both *in situ* and laboratory methods measure the fluorescence intensity of CDOM at the excitation and emission wavelengths of 370 and 460 nm. However, it must be noted that the two detailed values are uncomparable because the normalization method and unit are different for the two methods. CDOM concentration measured using *in situ* fluorescence sensor uses μg/L as the unit, but laboratory CDOM fluorescence uses the fluorescence intensity.

In addition, CDOM composition and source indices (*FI*_370_, HIX) were also strongly correlated with CDOM concentration measured using *in situ* fluorescence sensor (Figure 2c,d), indicating that CDOM concentration may be used to partially characterize CDOM sources and composition.

The comparison of CDOM concentration measured using *in situ* fluorescence sensor with the CDOM absorption and fluorescence of samples from a wide range of aquatic environments confirmed the strong positive relationship between CDOM concentration and absorption, fluorescence found in previous studies [10,21]. In addition, our study demonstrates that, making use of the *in situ* CDOM fluorescence sensor, this relationship was not affected by the presence of high total suspended matter (TSM) concentration, nearing 130 mg/L in the large, shallow lake. Previous studies presented different results regarding the effect of TSM concentration on CDOM concentration measured using *in situ* fluorescence sensor. Belzile *et al.* [21] reported that TSM did not interfere with the utility of CDOM fluorescence as a proxy for the CDOM absorption coefficient at 370 nm and DOC concentration in freshwater and coastal systems, suggesting the ability to make representative unfiltered measurements in a range of environments. However, Saraceno *et al.* [8] found that elevated TSM concentration could contribute significantly to the diffuse attenuation coefficient, potentially biasing a measurement intended only for CDOM during storm events. The contrasting results were attributed to a great difference in TSM concentration. The TSM concentration was as high as 1528.9 mg/L in the study by Saraceno *et al.* [8] but less than 35.0 mg/L in the study by Belzile *et al.* [21]. Our study further expanded the scope of CDOM sensor applications in turbid waters with TSM concentration less than 130 mg/L.

### 3.2. Spatial Distribution of CDOM Concentration

**Figure 3.**
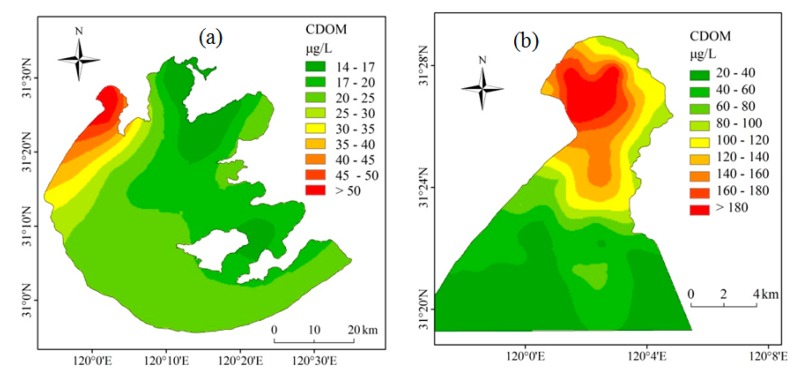
Spatial distribution of CDOM concentration measured using *in situ* fluorescence sensor in 2013 (**a**) and 2014 (**b**) in Lake Taihu.

Using the *in situ* fluorescence sensor, Figure 3 presents a large spatial variability in CDOM concentration in Lake Taihu, which was consistent with our previous study using laboratory CDOM absorption measurements [20]. Overall, the highest CDOM concentration was recorded in the northern Zhushan Bay, followed by Meiliang Bay and other lake regions (Figure 3). In Zhushan Bay, the CDOM concentration decreased from the inner bay to the outer regions and further to the open lake region. The spatial distribution of CDOM concentration was mainly controlled by the riverine input of organic matter from the northwest rivers [17,20]. Seasonally, CDOM concentration in Zhushan Bay in spring in 2014 was significantly higher than that in summer in 2013 (*t*-test, *p* < 0.001), which was consistent to our previous five years (2005–2009) long-term specific-site observation [20].

### 3.3. In Situ Fluorescence Sensor as a Proxy of DOC Concentration

**Figure 4.**
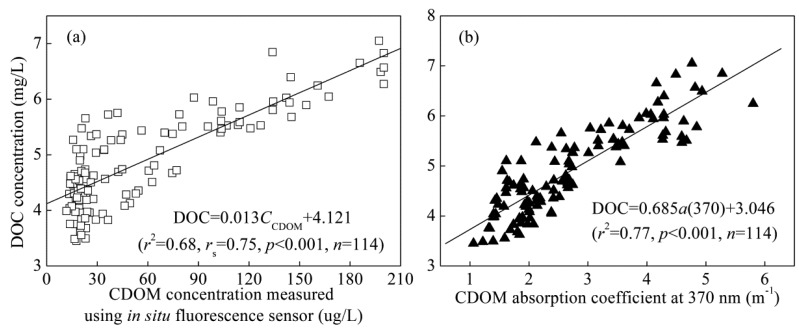
Linear relationships between dissolved organic carbon (DOC) concentration and CDOM concentration measured using *in situ* fluorescence sensor (**a**), laboratory CDOM absorption coefficient at 370 n (**b**) in Lake Taihu. *r* and *r*_s_ are the Pearson and Spearman correlation coefficients, respectively.

Although deviations in each measurement were observed, the general agreement between CDOM concentration measured using *in situ* fluorescence sensor, laboratory CDOM absorption coefficients and DOC concentration is highly significant (Figure 4), suggesting that CDOM concentration could be used as a proxy of DOC concentration in Lake Taihu. There was much more scatter in the relationship between DOC concentration and CDOM concentration measured using the *in situ* fluorescence sensor than in that between DOC concentration and laboratory CDOM absorption, resulting in a slight deterioration in the coefficient of determination (0.68 *vs.* 0.77). Considering that half of CDOM concentration is distributed in relatively low concentration (10–30 μg/L) and the Pearson correlation coefficient is skewed, the correlation was evaluated using the Spearman correlation coefficient. The Spearman correlation coefficient was 0.75, which was slightly lower the Pearson correlation coefficient of 0.82. However, the results showed that the correlation was still highly significant between DOC concentration and CDOM concentration measured using *in situ* fluorescence sensor (*p* < 0.001). Therefore, we think the application of *in situ* fluorescence sensor to predict DOC concentration is acceptable. The deterioration in the coefficient of determination was partly attributed to the presence of particles, which could impact fluorometer output during *in situ* deployment. In addition, the *in situ* fluorescence sensor is based on humic-like fluorescence intensity, but DOC concentration includes other protein-like fluorescent and non-fluorescent substances except for humic-like fluorescent substance, which might decrease the correlation.

In many coastal and lake waters where there is a significant source of allochthonous CDOM from the river and land and both CDOM optical properties and DOC concentration behave conservatively at local scales, it is possible to estimate DOC concentration with CDOM absorption through on-line continuous monitoring and remote sensing of CDOM. Based on these assumptions, significant empirical relationships between the CDOM absorption coefficient and DOC concentration have widely been observed in various waters [10,12,31–34]. For example, Kowalczuk *et al.* [10] recommended that CDOM concentration measured using *in situ* fluorescence sensor (TRIOS GmbH, Rastede, Germany) can be regarded as a DOC concentration proxy based on the measurement of thirteen research cruises in the Baltic Sea. Therefore, our result further confirmed that the relationship can be used to predict DOC concentration from both *in situ* fluorescence sensor and CDOM absorption coefficients of filtered water.

In comparison with correlations among CDOM concentration measured using *in situ* fluorescence sensor, absorption coefficients, fluorescence and composition parameters (Figure 2), the coefficient of determination was slightly low, indicating some seasonal variation of the optically inactive fraction of DOC (both non-absorbing and non-fluorescent) in Lake Taihu. In fact, some studies indicated that the optical properties of CDOM were not conservative and that they changed due to CDOM degradation processes such as photochemical and microbial degradation, which also lead to seasonal variability in CDOM absorption *vs*. DOC relationships in the coastal waters of oceans [35]. The data collected during the present study demonstrated that CDOM concentration measured using *in situ* fluorescence sensor could be used to observe DOC dynamics and as a proxy of DOC concentration with reasonable accuracy despite the seasonal variation in the optically inactive fraction of DOC.

### 3.4. Potential Application of in Situ Fluorescence Sensor for Real-time Monitoring of Water Quality

**Figure 5.**
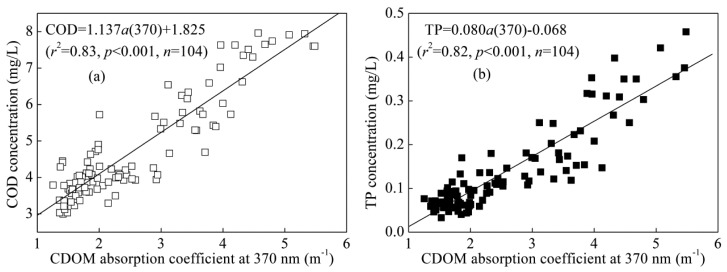
Linear relationship between CDOM absorption coefficient *a*(370) and the concentrations of chemical oxygen demand (COD) (**a**) and total phosphorus (TP) (**b**) in Lake Taihu.

To further expand the monitoring and surrogate measurements of water quality using a CDOM sensor, we analyze the correlations between CDOM absorption coefficients and TN, TP and COD concentrations in three different seasons (e.g., spring, summer and winter) in Lake Taihu. Because autumn is similar to spring in temperate and subtropical regions, autumn was not considered in this study. Highly significant linear relationships were found between *a*(370) and TP, COD concentrations (Figure 5). However, there was no significant correlation between CDOM absorption and TN concentration. The very tight correlation between the CDOM absorption coefficient and the *in situ* CDOM values (Figure 2a) and laboratory COD and TP concentrations (Figure 5) would suggest that robust correlations between CDOM concentration measured using *in situ* fluorescence sensor and laboratory COD and TP concentrations could also be obtained, at least at a regional scale.

COD represented the oxygen demand of dissolved matter, which is often used to characterize organic matter concentration and was an important lake water quality parameter representing the depletion of dissolved oxygen. However, there are several limitations of COD measurement in continuous monitoring [11]. For example, several hours are required for the completion of the COD measurements. The presence of toxic substances may influence the biochemical oxidation, resulting in analytical errors. Potassium dichromate, a typical oxidant for a COD test, cannot completely decompose organic matter in samples, and the degree of the chemical oxidation itself may be affected by organic matter composition and the molecular structures involved [36]. Therefore, it is necessary to develop more rapid and reliable monitoring techniques to replace the traditional COD measurements. Our results confirmed that CDOM concentration measured using *in situ* fluorescence sensor could be used to predict COD concentration, which was observed in other inland waters [11,22,23,37].

The success of predicting TP concentration using CDOM absorption coefficients may be attributed to similar trends in the variations in CDOM and phosphorus pollution and/or similar sources of the two types of pollution in the watershed. For example, the typical sources of CDOM and phosphorus pollution in Lake Taihu included the riverine input of northwest rivers. However, the failure to predict TN concentration using the CDOM absorption coefficient may be attributed to the strong seasonal variations in TN concentration with the maximum values occurring in winter and spring but minimum values appearing in summer and autumn [38]. When the correlation model was fit individually for three different seasons, there were significant linear relationships between *a*(370) and TN concentration in spring (*r*^2^ = 0.29, *p* < 0.001, *n* = 32), summer (*r*^2^ = 0.47, *p* < 0.001, *n* = 40) and winter (*r*^2^ = 0.75, *p* < 0.001, *n* = 32). Therefore, a seasonal predictive model should be developed if *in situ* CDOM sensor monitoring was used to predict TN concentration.

## 4. Conclusions

The strong correlations between CDOM concentration measured using an *in situ* fluorescence sensor and CDOM absorption, fluorescence and DOC concentration based on a large dataset confirmed the utility of an *in situ* CDOM sensor as a high-resolution CDOM absorption and DOC concentration proxy in eutrophic lakes. In addition, the highly significant correlations between laboratory CDOM absorption coefficients and COD and TP concentrations would further expand the potential application. However, the combination of *in situ* and laboratory measurements was necessary to develop site-specific relationships between fluorescence sensor and DOC, COD and TP concentrations because the relationships between the measured variables in our study were not general for all natural waters but for specific waters. Using the *in situ* fluorescence sensor as the proxy, the accurate, high-resolution time series of water quality parameter dynamics could be established. *In situ* CDOM measurements are particularly useful during algal blooms and black water events, especially in drinking water sources where high-frequency measurements are necessary to capture rapid changes in water quality and provide an early warning signal for the water supply. The high-frequency *in situ* CDOM and dissolved oxygen monitoring combined with an early warning system would avoid the drinking water crisis that occurred in 2007 [3]. In addition, *in situ* CDOM observations are especially useful in monitoring CDOM dynamics related to anthropogenic activities, such as rapid changes in land use and industrial spills of organic matter. Therefore, acquisition of high-frequency, long-term data series or high-resolution profiles is feasible and should help increase our understanding of biogeochemical processes and ecosystem responses in natural waters. In addition, continuous water quality monitoring is essential for efficient management of lake water quality and for the prompt control of pollution.
